# Building digital bridges: Sustaining medical education in Ukraine during the war through blended online modules

**DOI:** 10.3205/zma001861

**Published:** 2026-06-15

**Authors:** Sarah König, Nina Luisa Zerban, Nataliia Malachkova, Joy Backhaus, Halyna Rudenko

**Affiliations:** 1 University Hospital Würzburg, Institute of Medical Teaching and Medical Education Research, Würzburg, Germany; 2 University Hospital Würzburg, Centre for Study Programme Management and Development, Würzburg, Germany; 3 Julius-Maximilians University of Würzburg, Office of the Dean of Studies, Würzburg, Germany

**Keywords:** blended learning, medical education, distance learning, perceived outcome dimensions, communication skills, clinical reasoning, Ukraine

## Abstract

**Objectives:**

The invasion of Ukraine has severely disrupted medical education. This study evaluated a blended digital intervention to support medical students and examined factors influencing students’ experiences under the conditions.

**Methods:**

Two blended online modules (Medical Communication and Clinical Reasoning) were co-developed by the University of Würzburg and two Ukrainian medical universities. The modules combined asynchronous learning materials with synchronous online sessions, including simulated patients alongside case-based discussions. Displaced Ukrainian physicians contributed as instructors. A cross-sectional mixed-methods evaluation was conducted at the end of teaching using an online questionnaire with 15 Likert-scale items and open-ended questions. Quantitative data were analysed using descriptive statistics, exploratory factor analysis, and regression analyses with interaction terms. Qualitative responses underwent thematic content analysis.

**Results:**

In total, 376 valid questionnaires were completed (response rate: 28.8% of 1,306 module participations). Factor analysis identified three perceived outcome dimensions: teaching quality, learning gain, and module experience. These accounted for 54% of the total variance and captured students’ perceived effectiveness of the modules. Ratings were high for organisation, clarity, and interactivity, while perceived learning gain was positive but lower for compensating missing practical training. War- and infrastructure-related interruptions were negatively associated with ratings, with a significant interaction of study location indicating stronger effects in Ternopil than in Vinnytsya. Qualitative comments corroborated these findings and highlighted practical relevance, cultural adaptation, and participants’ exposure to different professional practices.

**Conclusion:**

The blended online modules were positively evaluated, and represent a scalable and resilient approach to sustaining medical training in conflicts and other resource-limited contexts.

## Zusammenfassung

**Ziel:**

Die Invasion der Ukraine hat die medizinische Ausbildung erheblich beeinträchtigt. Ziel dieser Studie war es, eine digitale Blended-Learning-Intervention zur Unterstützung von Medizinstudierenden zu evaluieren und Einflussfaktoren auf die studentischen Erfahrungen unter diesen Bedingungen zu untersuchen.

**Methoden:**

Zwei Blended-Online-Module („Ärztliche Kommunikation“ und „Klinische Entscheidungsfindung“) wurden gemeinsam von der Universität Würzburg und zwei ukrainischen medizinischen Universitäten entwickelt. Die Module kombinierten asynchrone Lernmaterialien mit synchronen Online-Sitzungen, einschließlich Simulationspersonen und fallbasierter Diskussionen. Geflüchtete ukrainische Ärztinnen und Ärzte wirkten als Lehrende mit. Am Ende der Module wurde eine querschnittliche Mixed-Methods-Evaluation mittels eines Online-Fragebogens durchgeführt, der 15 Likert-skalierte Items und offene Fragen umfasste. Die quantitativen Daten wurden deskriptiv sowie mittels explorativer Faktorenanalyse und Regressionsanalysen mit Interaktionstermen ausgewertet. Die qualitativen Antworten wurden inhaltsanalytisch aufbereitet.

**Ergebnisse:**

Insgesamt wurden 376 gültige Fragebögen ausgewertet (Rücklaufquote: 28,8 % von 1.306 Modulteilnahmen). Die Faktorenanalyse identifizierte drei Ergebnisdimensionen: Lehrqualität, Lernzuwachs und Modulbewertung. Diese erklärten 54 % der Gesamtvarianz und bildeten die wahrgenommene Effektivität der Module ab. Die Bewertungen fielen insbesondere hinsichtlich Organisation, Verständlichkeit und Interaktivität hoch aus. Der wahrgenommene Lernzuwachs wurde ebenfalls positiv bewertet, jedoch geringer im Hinblick auf die Kompensation fehlender praktischer Ausbildung. Kriegs- und infrastrukturbedingte Unterbrechungen waren negativ mit den Bewertungen assoziiert; eine signifikante Interaktion mit dem Studienort zeigte stärkere Effekte in Ternopil im Vergleich zu Vinnytsya. Die qualitativen Rückmeldungen bestätigten diese Ergebnisse und hoben darüber hinaus die praktische Relevanz, die kulturelle Anpassung sowie Einblicke in unterschiedliche berufliche Praxis hervor.

**Schlussfolgerung:**

Die Blended-Online-Module wurden insgesamt positiv bewertet und stellen einen skalierbaren und resilienten Ansatz zur Sicherung medizinischer Ausbildung in Konflikt- und ressourcenlimitierten Kontexten dar.

## 1. Introduction

The war against Ukraine, which began in February 2022, has severely disrupted medical education in the country [1]. Damage and destruction of infrastructure forced many faculty members at medical universities and hospitals to flee their hometowns or the country, or to be redeployed to frontline care for wounded soldiers and civilians. As a result, medical students have struggled to complete their studies, with in-person teaching and clinical training becoming increasingly difficult or even impossible [2]. In this context, the Russian invasion also necessitated a rapid transition to virtual learning to sustain medical education [3]. While this shift preserved access, it exposed gaps in practical, communication, and clinical reasoning skills [4]. At the same time, many medical students were also displaced within Ukraine or across Europe. 

In response, the Faculty of Medicine at the University of Würzburg implemented the “Ukraine Medical Satellite Teaching” (UA-MEDSAT) project. The project aimed to mitigate the impact of the war on Ukrainian medical education by providing targeted blended online modules to address curricular gaps and ensure students could continue their studies [5]. Encouragingly, a meta-analysis indicated that well-structured online formats can complement traditional instruction and significantly improve communication skills and knowledge acquisition [6].

The project introduced two modules: “Module 1: Medical communication and counselling skills” and “Module 2: Clinical reasoning and case based discussions” for medical students at two partner universities in Ukraine: I. Horbachevsky Ternopil National Medical University (TNMU) and National Pirogov Memorial Medical University Vinnytsya (VNMU). Using a blended learning approach that combined asynchronous digital content with synchronous online sessions, the initiative enabled flexible, location-independent learning that accommodated students displaced by the war or otherwise unable to attend traditional classes. Emphasis was placed on communication skills, clinical reasoning, and case-based learning, with innovative elements such as live simulated patients integrated to create interactive and practical learning experiences. Such competencies had previously received limited attention in Ukrainian medical curricula, and the project sought to strengthen and expand these areas.

The UA-MEDSAT project actively employed Ukrainian medical professionals displaced by the war and residing in Germany in teaching and content development, ensuring academically robust and culturally as well as linguistically appropriate modules. The programme also enabled Ukrainian medical students to continue their studies and retain access to clinically relevant educational input despite the severe disruption. Conceptually, the project was grounded in principles of blended learning and resilient, crisis-responsive medical education aimed at sustaining educational quality and learner engagement under the conditions resulting from the crisis [7], [8].

Against this background, the evaluation focused on the following key questions:

- How effective are the blended online modules in terms of students’ perceived teaching quality, learning gain, and overall module experience in clinically oriented communication and clinical reasoning teaching?
- Do students’ perceptions differ between the two study locations?
- To what extent can war- and infrastructure-related interruptions explain observed location differences?

These questions were examined using a cross-sectional mixed-methods educational evaluation designed to assess the project’s implementation and its potential as a model for medical education in a volatile environment affected by war and conflict.

## 2. Methods

### 2.1. Context of the UA-MEDSAT project

The Faculty of Medicine at the University of Würzburg collaborated with the two Ukrainian medical universities TNMU and VNMU to provide blended online modules for medical student participants from September 1 to December 31, 2022. The UA-MEDSAT project was financially supported by the German Academic Exchange Service (DAAD) within the framework of the funding programme "Ukraine digital".

Teaching was conducted by Ukrainian physicians and trained amateur actors serving as simulated patients (SPs), many of whom had fled to Germany to escape the invasion. This enabled teaching in Ukrainian and provided employment for displaced staff. All staff involved received targeted training in content delivery and didactic methods to ensure effective teaching. Existing teaching modules and course concepts from the Faculty of Medicine at Würzburg were adapted in close collaboration with Ukrainian lecturers to align with the specific needs of Ukrainian medical curricula and the requirements of distance learning. 

Two online blended modules were designed to be interactive and included practical elements to compensate, as much as possible, the lack of direct patient contact during training. The teaching approach integrated asynchronous digital resources with live synchronous sessions conducted via video conference, utilizing breakout rooms to facilitate small-group, interactive learning. The Ukrainian partner universities integrated the blended online modules into their curricula by designating specific time slots for the sessions and aligning the content with appropriate semesters. In Ternopil, participation was part of an existing communication skills course, whereas in Vinnytsya, it was offered as an additional activity within allocated timetable slots.

### 2.2. Design and structure of the blended online modules

The UA-MEDSAT project offered two blended modules to prepare students for patient-centred care. Module 1, “Medical Communication and Counselling Skills”, enhanced communication abilities through a blended learning approach. In the asynchronous phase, students accessed digital resources on communication strategies, role-play, and best-practice videos. All materials were available in Ukrainian. The module covered patient interaction, history-taking, and delivering bad news. Skills were refined in synchronous sessions with feedback, peer role-play, and small-group discussions. Simulated patients were used in telemedicine exercises. Six online sessions (2 hours each) were held in groups of about 12 students. Module 2, “Clinical Reasoning and Case-Based Discussions”, strengthened clinical reasoning. Students analysed clinical data and developed diagnoses and management plans through shared decision-making. After preparatory work, they joined these case-based discussions in five disciplines: surgery (4 sessions), internal medicine/therapy (2), gynaecology/obstetrics (2), paediatrics (2), and neurology (2). Slides came from Würzburg and Erlangen University Hospitals and were translated into Ukrainian. The module comprised 12 seminars (two hours each) with groups of 22–25 students.

### 2.3. Evaluation of the blended online modules

Data were collected using a single survey instrument comprising both quantitative and qualitative components. The questionnaire included structured items assessing students’ perceptions as well as open-ended questions to capture qualitative feedback and was administered at the end of the teaching period. Both data strands were integrated at the interpretation level to provide a complementary understanding of the findings.

The general section of the questionnaire collected demographic information (gender, age, semester), study location, devices used to access the courses, and the frequency of interruptions. Interruptions included air-raid alerts (civil defence sirens warning of imminent aircraft, missile, or drone attacks) and technical failures (internet outages and power supply shortages) and were rated on a five-point Likert scale ranging from none (1) to very frequent (5). For analysis, these responses were grouped into infrequent (1-3) and frequent (4-5) interruptions.

For the module-specific evaluation, the questionnaire was specifically developed for this project and does not correspond to a single published standardised instrument. Instead, it represents a pragmatic, theory- and literature-based item set drawing on the authors’ experience in course evaluation. Questionnaire development was guided by commonly described dimensions of educational evaluation in medical education, encompassing educational structure, educational processes, learning outcomes, and complemented by the systematic use of evaluation results for continuous quality improvement [1]. 

A team of three medical education experts developed and refined the items through pre-testing, resulting in a final 15-item questionnaire in German. This version was subsequently translated into Ukrainian by two independent bilingual translators. The translations were cross-checked and reconciled to ensure conceptual accuracy and linguistic clarity. All items were rated on a five-point Likert scale (1=strongly disagree to 5=strongly agree) and complemented by an open-ended section for qualitative feedback. 

Exploratory factor analysis (EFA) was conducted to examine the underlying dimensional structure of the item set. The analysis aimed to explore whether coherent latent dimensions reflecting different aspects of students’ evaluations of perceived outcomes could be identified. No objective performance measures were collected.

### 2.4. Statistical analysis

All statistical analyses were conducted using Microsoft Excel (Microsoft Corporation, Redmond, WA, USA; Version 16.70, 2023) and R statistical software (R Core Team, Vienna, Austria; version 4.4.2). Descriptive statistics included the mean (M), standard deviation (SD), minimum (Min), maximum (Max), and skewness (skew). To examine the psychometric properties of the questionnaire, a maximum likelihood EFA with promax rotation was performed [2]. Suitability for EFA was determined using Bartlett’s test of sphericity and the Kaiser-Meyer-Olkin (KMO) coefficient. The KMO coefficient measures shared variance among items, and Bartlett’s test evaluates the null hypothesis that items were not correlated [3]. The factor solution was deemed valid if it met the following criteria: Bartlett’s test of sphericity (p<0.05), KMO coefficient >0.50, Eigenvalues >1, factor loadings >0.30 without double loadings, and communalities >0.40 [4]. The number of factors was determined using a combination of scree plot inspection, parallel analysis, and critical evaluation of factor interpretability [5], [6]. Internal consistency was determined by calculating Cronbach’s alpha values (a), with values exceeding 0.7 considered acceptable and those above 0.8 classified as good [7].

Regression analyses were conducted to estimate standardized beta coefficients (β) while controlling for relevant covariates [8], [9]. Interaction effects were examined by including interaction terms in the regression models to assess potential changes in effect direction and statistical significance. As no a priori moderation hypotheses were specified; interaction terms were included for exploratory purposes to examine conditional associations. For each model, explained variance was reported as R^2^, along with the corresponding standard error (SE). Differences between study locations were analysed using the factor-based scales. Interruption frequency was included as the primary covariate, as no additional variables could be assessed reliably under the given conditions. 

### 2.5. Qualitative analysis of open-ended responses

Following an inductive approach, all comments were read in full, coded, and iteratively grouped into recurring thematic categories [10]. Coding consistency was ensured through repeated comparison and refinement with two members of the research team. The frequency of each theme was recorded to indicate its prevalence in the dataset. To illustrate participants’ perspectives, representative paraphrased statements were selected for each theme. This approach complemented the quantitative results and provided additional insight into participants’ experiences.

## 3. Results

### 3.1. Demographics and study context of participants

Table 1 (Tab. 1) summarises the characteristics of the participating students. Students participated either in module 1 or module 2; due to the curricular allocation of the courses, participation in both modules was not possible. Two students who nevertheless indicated having participated in both modules were excluded from the analysis. The final sample comprised n=376 completed questionnaires, corresponding to a response rate of 28.8% based on 1,306 recorded and valid module participations. Slightly more respondents were from Vinnytsya. Furthermore, module 1 received more evaluations than module 2. Most participants were female, in their early twenties, and students from Vinnytsya were, on average, further advanced in their studies. Laptops and smartphones were the predominant devices to access the courses. Frequent interruptions were reported more often in Ternopil (84.3%) than in Vinnytsya (65.1%). 

### 3.2. Student feedback on module effectiveness

Table 2 (Tab. 2) presents the descriptive statistics for the 15-item module-specific evaluation questionnaire. Responses utilised the full range of the Likert scale (1-5), and all items demonstrated negative skew (-2.71 to -0.91), demonstrating a general trend of agreement. The module-specific evaluation revealed generally positive feedback, with mean scores ranging from 3.87 to 4.71. Participants rated the organization, logical structure, and alignment with prior knowledge highly. Teaching delivery, including clarity, practical examples, and responsiveness to questions, was well-received, as were the learning materials. Active participation and a positive group atmosphere were notable strengths. While participants reported positive educational benefits and valued insights into German medical practices, the perceived ability of the modules to compensate for missing practical training was rated lower. 

### 3.3. Underlying dimensions of blended online modules

Three dimensions were extracted from the questionnaire data (see table 3 (Tab. 3)), explaining 54% of the total variance prior to rotation in students’ responses across the 15 questionnaire items. Sampling adequacy was excellent (KMO=0.95), and Bartlett's test of sphericity was significant (χ^2^=4366.64, df=120, p<0.001). 

As indicators of perceived effectiveness, students’ subjective evaluations were reported across three outcome dimensions: teaching quality (8 items, α=0.89), focusing on clarity of delivery, learning materials, and teacher responsiveness; learning gain (3 items, α=0.82), capturing perceived knowledge improvement and the compensatory value of the course; and module experience (4 items, α=0.89), reflecting organization and enjoyment. Internal consistency was high, with communalities ranging from 0.28 to 0.86. Scale scores correlated strongly (r=0.75-0.85, p<0.001). 

### 3.4. Differences between study locations

**Figure 1:**
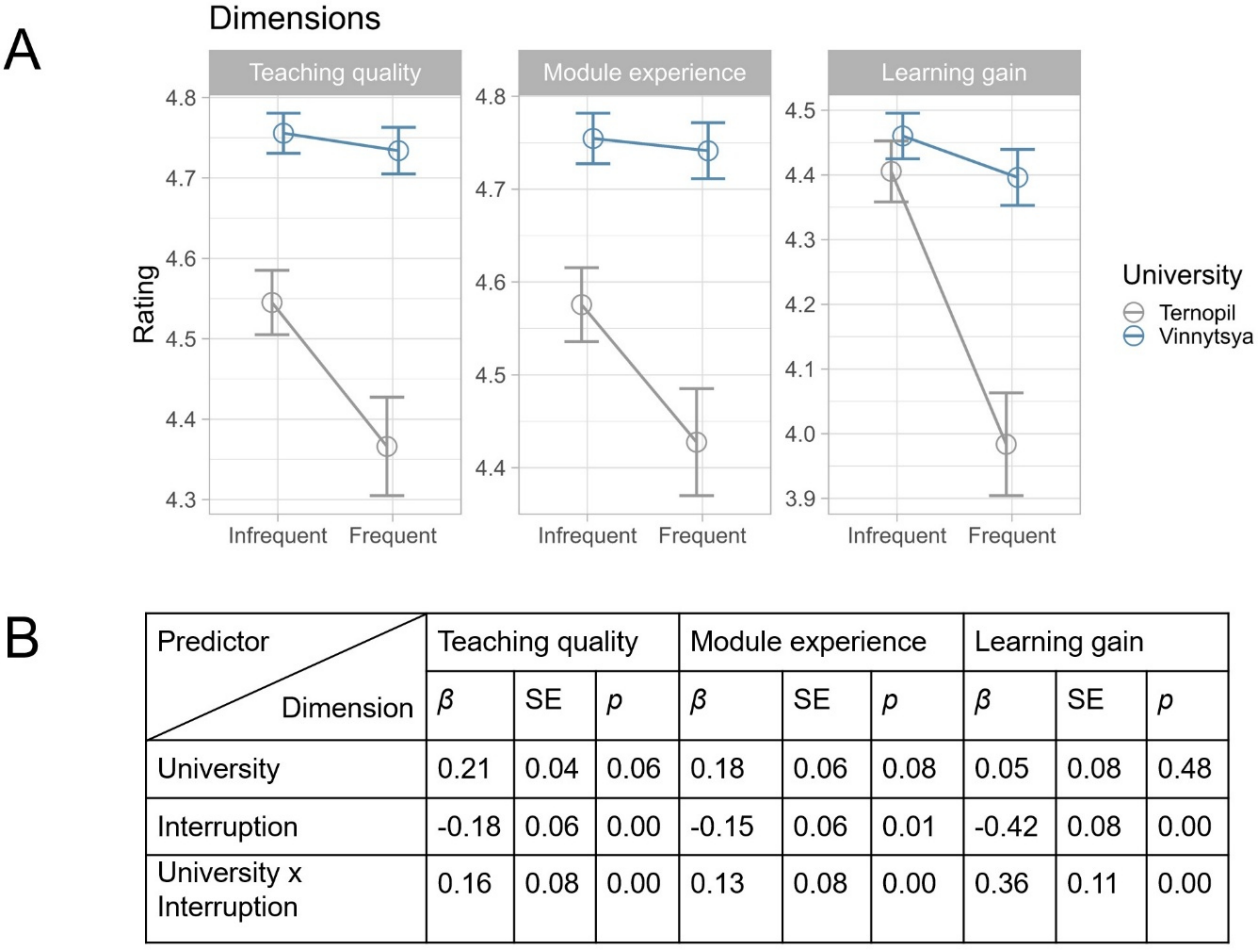
Impact of interruption frequency on perceived outcome dimensions by study location (A) Mean ratings for teaching quality, module experience, and learning gain in Ternopil and Vinnytsya, stratified by interruption frequency (infrequent vs. frequent). Points indicate mean values, error bars indicate 95% confidence intervals. (B) Results of regression analyses illustrating the main effects of interruption frequency and study location (university), as well as their interaction, on the three outcome dimensions. Standardized β-coefficients, SE, p values, and model R^2^) are reported, p values <0.05 were considered statistically significant.

Differences between study locations were analysed across the three perceived outcome dimensions. Among the participant characteristics reported in table 1 (Tab. 1), interruption frequency was the only variable associated with the dimensions, whereas no associations were observed for demographic or study-related characteristics. Figure 1 A (Fig. 1) illustrates the relationship between interruption frequency and the three outcome dimensions separately for Ternopil and Vinnytsya. Overall, ratings tended to be higher in Vinnytsya. When interruptions were reported as infrequent, ratings for teaching quality, learning gain, and module experience were more similar between the two locations. In Ternopil, frequent interruptions were associated with lower ratings across all three dimensions, with the most pronounced decrease observed for learning gain, whereas ratings in Vinnytsya displayed comparatively little variation by interruption frequency. These descriptive patterns were confirmed by regression analyses (see figure 1 B (Fig. 1)), with all reported β coefficients representing standardized effects. A positive but non-significant effect for study location was observed across all three outcome dimensions (β ranging from 0.05 to 0.21), accounting for 6%-8% of explained variance. A negative main effect for interruptions was observed across all outcome dimensions (β ranging from -0.18 to -0.42). This effect was most pronounced for learning gain (β=-0.42, R^2^=0.11), followed by teaching quality (β=-0.18, R^2^=0.08) and module experience (β=-0.15, R^2^=0.07), explaining between 7%-11% of the variance across outcomes. In addition, significant interaction effects between study location and interruptions were identified for all three dimensions, with positive interaction coefficients for learning gain (β=0.36, R^2^=0.09), teaching quality (β=0.16, R^2^=0.07), and module experience (β=0.13, R^2^=0.07).

At the item level, this overall pattern was not consistently observed across all aspects of the perceived learning gain dimension. No significant location-related differences were found for the items “Compared to my previous knowledge, I learned a lot” and “The online courses help to compensate for the lack of practical training”, indicating stable ratings for these aspects across locations.

### 3.5. Qualitative feedback from open-ended responses

Of the 376 valid questionnaires, 298 participants (79.3%) provided at least one open-ended comment. In line with the quantitative findings, thematic analysis demonstrated that most remarks aligned with the high ratings of the modules, particularly with regard to perceived relevance, clarity of instruction, and the interactive case-based format (see table 4 (Tab. 4)). In consistence with high scores for the perceived dimensions of teaching quality and learning gain, many students highlighted improvements in communication skills and valued the opportunities to work with simulated patients. Qualitative comments also contextualised the quantitative findings related to intercultural learning with positive references to insights into German healthcare practices and the adaptation of content to the Ukrainian context. Conversely, themes related to challenges helped to explain lower ratings observed in specific settings. These included mentions of air-raid alerts and technical failures, as well as suggestions for longer practice opportunities or access to session recordings. 

## 4. Discussion

The UA-MEDSAT project addressed key challenges in Ukrainian medical education during the early stages of the current phase of the Russo-Ukrainian War and demonstrates the potential of flexible, culturally sensitive distance learning under conditions of crisis [11]. Overall, the evaluation findings indicate that this project format was feasible and well accepted. At the same time, the results reflect students’ perceived effectiveness of the blended online modules rather than objective learning outcomes or clinical performance. The integration of quantitative ratings and qualitative feedback revealed a consistent pattern: high ratings for teaching quality and module experience were mirrored by qualitative comments highlighting clarity of instruction, relevance of content, and the use of interactive case-based formats, while war- and infrastructure-related interruptions provided a plausible explanation for lower ratings in the specific context.

### 4.1. High levels of satisfaction with blended online modules

Students reported high satisfaction with teaching quality, organization, and engagement opportunities, consistent with evidence for blended learning in medical education [12], [13]. Practical, interactive elements such as SP courses mirrored successful COVID-19 era approaches, including telemedicine-based practice interviews [14], [15]. Similar findings from Ukraine have been reported that even in wartime, students value flexible formats that sustain motivation and performance [16]. By being implemented at the very beginning of the war, the project helped students avoid the sense of losing the opportunity to acquire essential competencies in patient communication and clinical reasoning. In this way, it facilitated a smoother educational transition from the acute phase of crisis towards a gradual return to a more stable learning routine during the subsequent war years. Module 1’s role-play and structured feedback supported empathy and patient-centred care [17]. Module 2’s case-based discussions promoted collaborative problem-solving, confirming that active participation in peer discussions is essential for developing diagnostic reasoning [18]. 

### 4.2. Impact of interruptions on perceived outcome dimensions

While the blended online modules were highly rated overall, interruptions, such as air-raid alerts and technical failures, significantly disrupted learning, particularly in Ternopil. Accordingly, location differences were largely explained by interruption frequency, which emerged as the primary interacting factor, while no substantial contribution by other contextual variables could be identified. Air-raid alerts, often at night and in the mornings, coincided with the morning teaching schedule present in Ternopil. In contrast, Vinnytsya's afternoon modules faced fewer interruptions, resulting in a more stable learning environment. These findings are consistent with Ukrainian reports highlighting the adverse impact of unstable learning environments [11], [19]. The findings also echo observations from other Ukrainian institutions during the war, in which the unpredictability of external threats posed significant organizational and pedagogical challenges to (distance) learning [12]. In this study, frequent interruptions reduced ratings for the dimension learning gain, showing that even well-designed modules cannot fully compensate for such interruptions. Ratings in Vinnytsya remained relatively stable, underscoring the importance of resilient delivery systems. The disparity between locations underscores the need for contingency planning and robust infrastructure to sustain education in conflict zones.

### 4.3. Insights into the German healthcare system

One of UA-MEDSAT’s strengths was the intercultural dimension of the modules, which provided participants with insights into the German healthcare system. This intercultural exchange, defined as structured exposure to the different healthcare system, communication practices, and clinical approaches, enriched the learning experience and supported participants’ professional development in an increasingly globalised medical context [20]. Through collaboration with German lecturers, students and faculty from the Ukrainian partner universities were exposed to different approaches to patient-centred communication, clinical decision-making, and advanced treatment strategies. In this context, the emphasis on patient-centred communication and shared decision-making aligns with established principles of German medical education [21] and reflects competencies that are widely regarded as essential for effective healthcare delivery, particularly in diverse and multicultural settings [22]. In addition, the integration of telemedicine into the curriculum aligns with current educational initiatives promoting digital health competencies as core skills for medical graduates, exemplified by the successful implementation of a digital health modules [23].

### 4.4. Opportunities for Ukrainian faculty and simulated patients

The project provided meaningful opportunities for Ukrainian faculty and simulated patients involved in teaching. For displaced Ukrainian professionals, participation enabled continued professional engagement, supported integration into the German healthcare context, and helped maintain clinical and educational competencies. This illustrates a viable model for leveraging the expertise of displaced professionals within educational initiatives during situations of crisis [24]. Moreover, the use of simulated patients, trained and deployed in a telemedicine format, enhanced the realism in online teaching. This approach is consistent with established frameworks in healthcare simulation, which highlight the importance of realistic scenarios in developing practical skills and professional competencies [25].

### 4.5. Limitations and areas for improvement

The project was implemented within a short timeframe, less than a year after the outbreak of the current phase of the war in Ukraine, and in a situation of considerable uncertainty. The ongoing conflict, migration of faculty and students, and disruption of educational systems required a pragmatic organizational approach. Participation formats varied, with some students attending voluntarily and others as part of a modified curriculum. As participation in the evaluation was voluntary, a selection bias towards more motivated students cannot be excluded.

A further limitation was the lack of longitudinal follow-up, which prevented an assessment of whether the skills and knowledge gained were successfully transferred into clinical practice. Future initiatives would benefit from incorporating follow-up evaluations to determine the long-term impact on professional development and patient care.

The situation of crisis also created substantial technical and logistical challenges. Frequent interruptions from missile strikes and air-raid alerts, combined with unreliable internet access, repeatedly disrupted learning, even though lecturers made concerted efforts to adapt by spontaneously rescheduling sessions or continuing teaching units at a later time whenever conditions allowed. These challenges underline the importance of investing in resilient technological infrastructure and developing robust contingency plans to safeguard the continuity of online education in similarly unstable environments.

## 5. Conclusion

UA-MEDSAT demonstrated that medical education can be sustained through innovative, culturally adapted, and resilient online teaching. By integrating displaced medical professionals into content delivery and combining asynchronous resources with interactive live sessions, the programme maintained learner engagement and achieved strong ratings across the outcome dimensions teaching quality, learning gain, and overall experience, even under severe external disruptions. The pronounced impact of air-raid alerts on learning gain underscores the need for contingency planning and robust infrastructure in crisis settings. UA-MEDSAT provided a transferable model for delivering clinical education under extreme conditions and highlights the value of international collaboration in safeguarding the training of future healthcare professionals. Its positive evaluation further illustrates the potential to sustain medical training and foster intercultural exchange in both conflict zones and other resource-limited contexts.

## Acknowledgements

We would like to express our deepest gratitude to all the students who participated in this study. Furthermore, we would like to thank Andrew Entwistle for his assistance with proofreading the manuscript.

## Notes

### Author contributions

SK, NZ, and HR were responsible for the development of the questionnaire, the conception and execution of the study, as well as drafting and revising the manuscript. JB was in charge of the statistical evaluation and played a key role in both drafting and revising the results. NZ assisted in evaluating the online courses and in administering the survey. HR implemented the courses, taught the courses with colleagues from Ukraine, and contributed to data collection. All authors approved the submitted manuscript and accept responsibility for their individual contributions as well as for ensuring that any issues concerning the integrity of the work are addressed appropriately.

### Authors’ ORCIDs

- Sarah König: [0000-0003-4866-9881]
- Nina Luisa Zerban: [0009-0003-7122-2946]
- Nataliia Malachkova: [0000-0002-7899-379X]
- Joy Backhaus: [0009-0005-6166-8973]

### Funding

The German Academic Exchange Service funded the project “UA-MEDSAT: Ukraine MEDical SAtellite Teaching” within the framework of “Ukraine digital: Ensuring academic success in times of crisis (2022)”.

### Data availability

The dataset utilized in this study is available from the corresponding author on request.

### Ethical approval

The institutional review and ethics board in Würzburg judged the project as not representing medical or epidemiological research on human subjects and that no ethical approval was required according to the Declaration of Helsinki. Thus, a simplified assessment protocol was adopted. The project was approved without any reservation under the proposal number 20221108 02. The student survey was conducted anonymously, and participation in the evaluation was voluntary. 

## Competing interests

The authors declare that they have no competing interests. 

## Figures and Tables

**Table 1:**
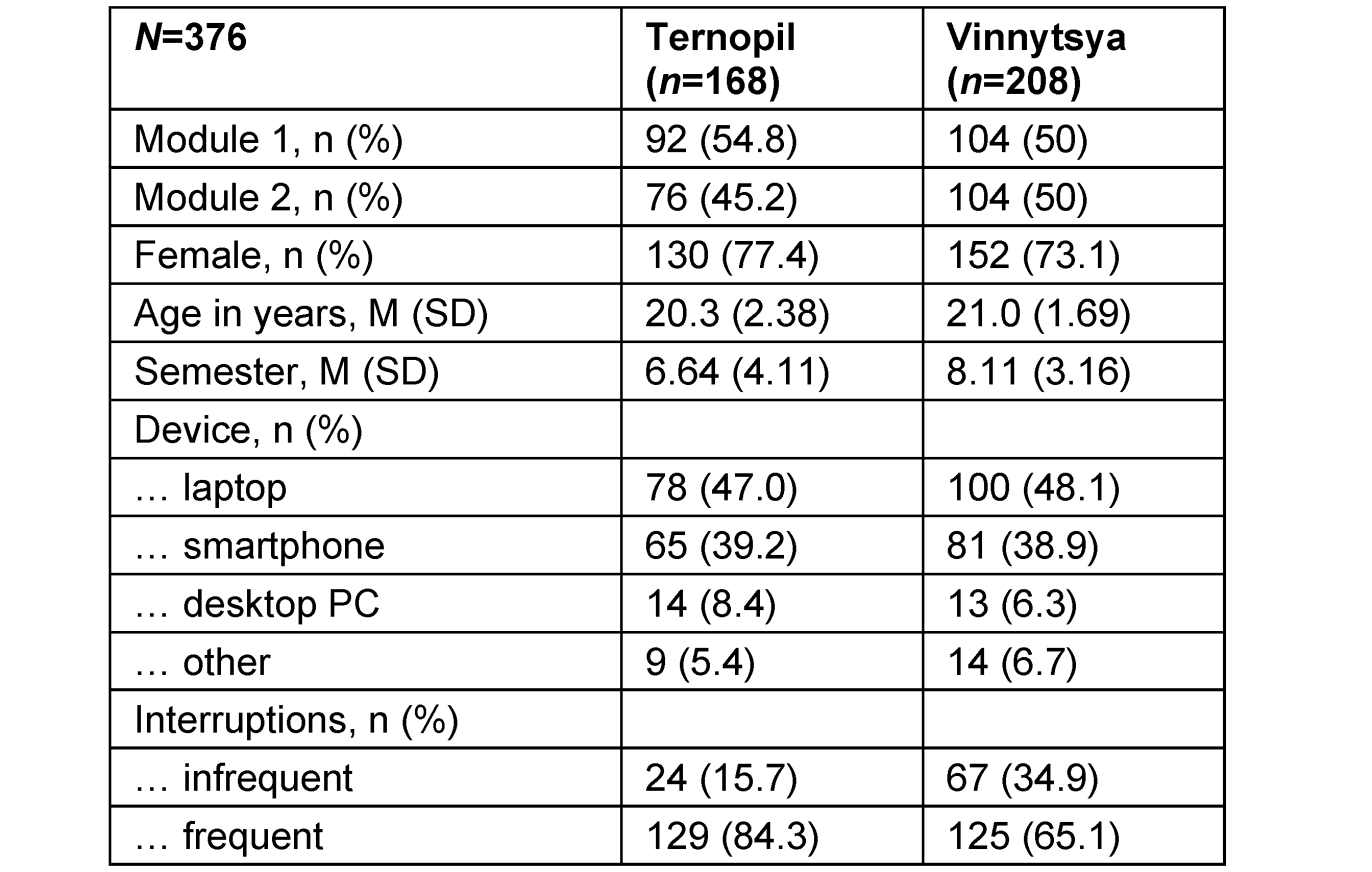
Characteristics of participating students

**Table 2:**
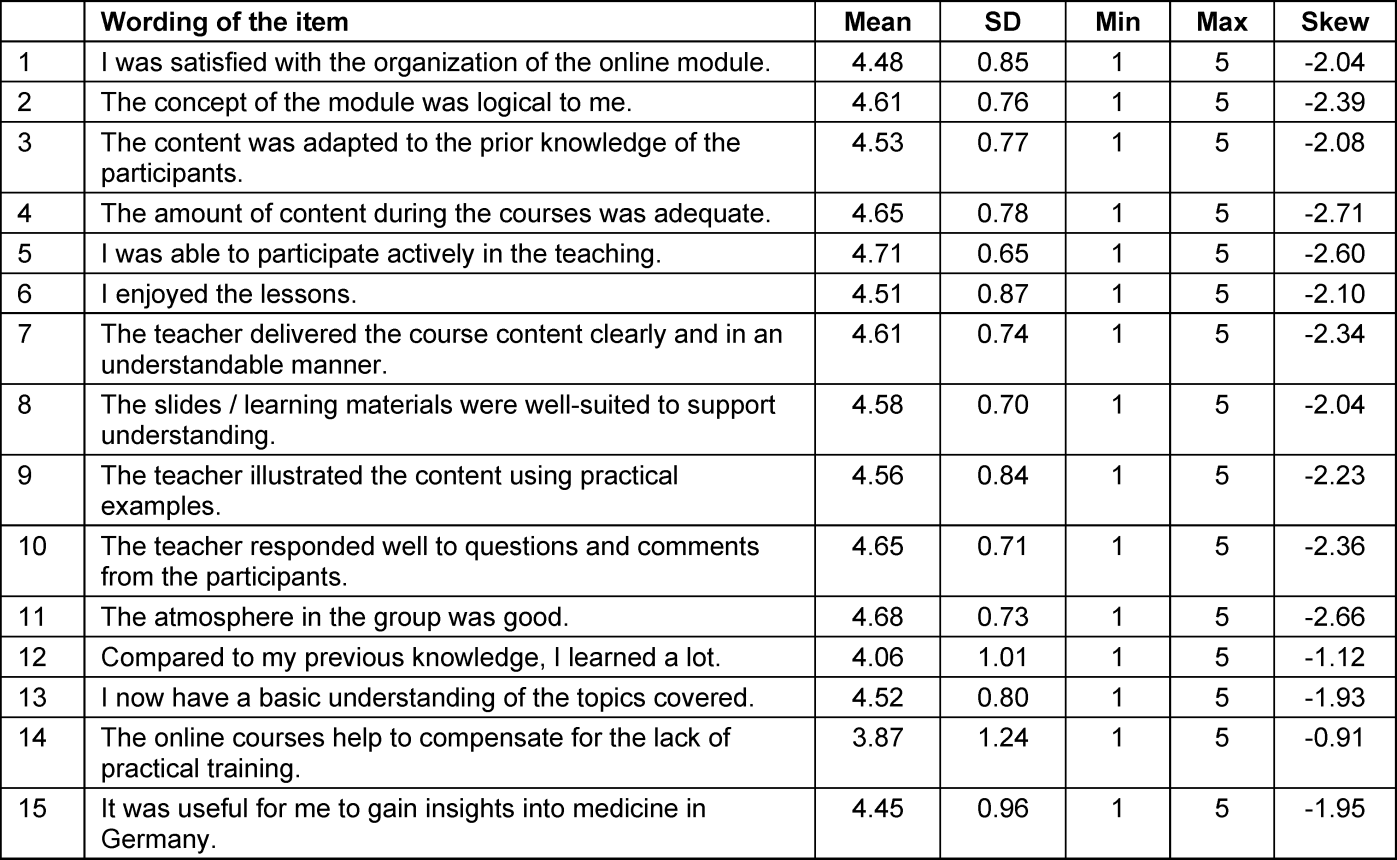
Descriptive statistics of the items from the blended online module-specific evaluation

**Table 3:**
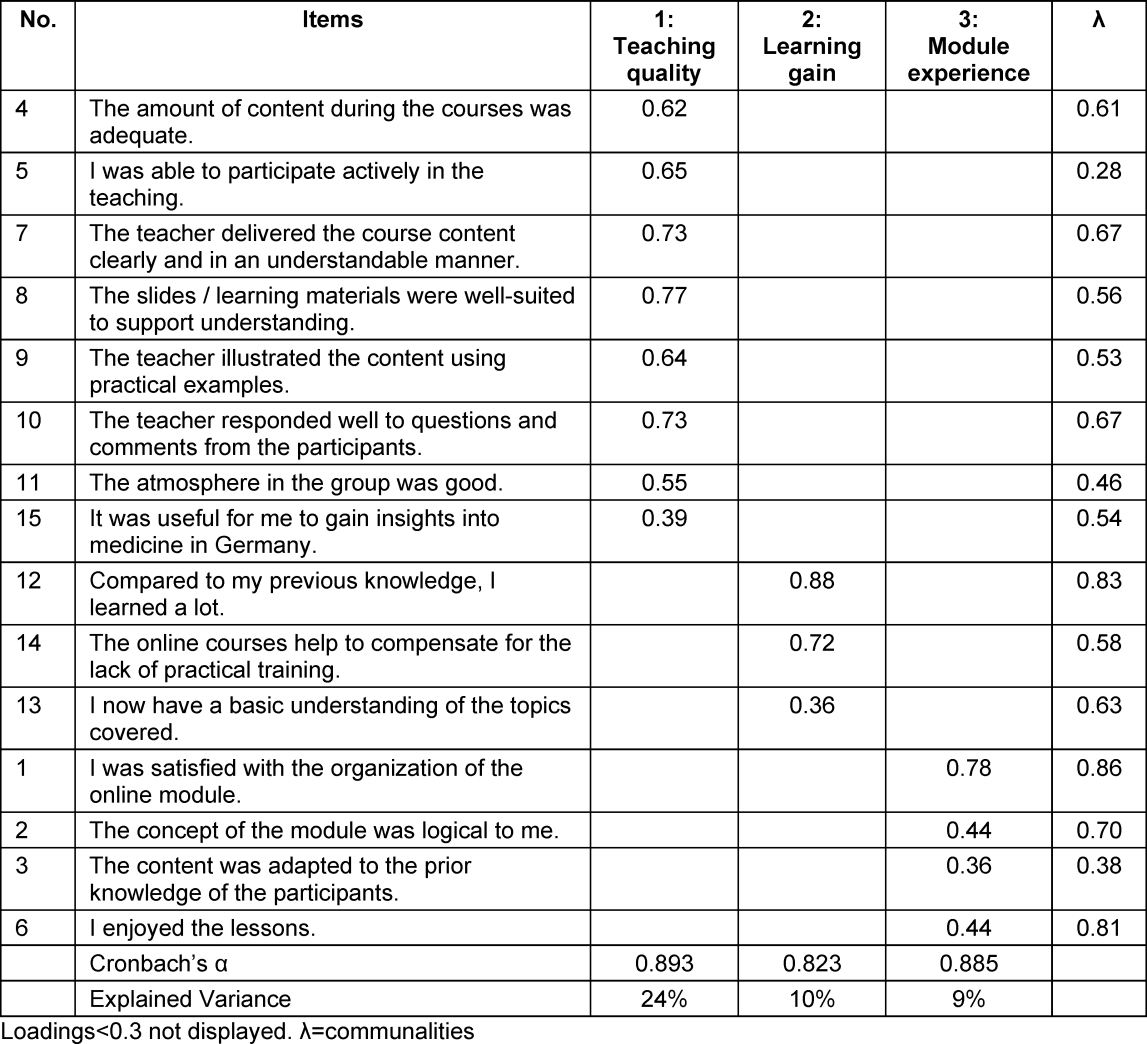
EFA for the questionnaire items and identified outcome dimensions

**Table 4:**
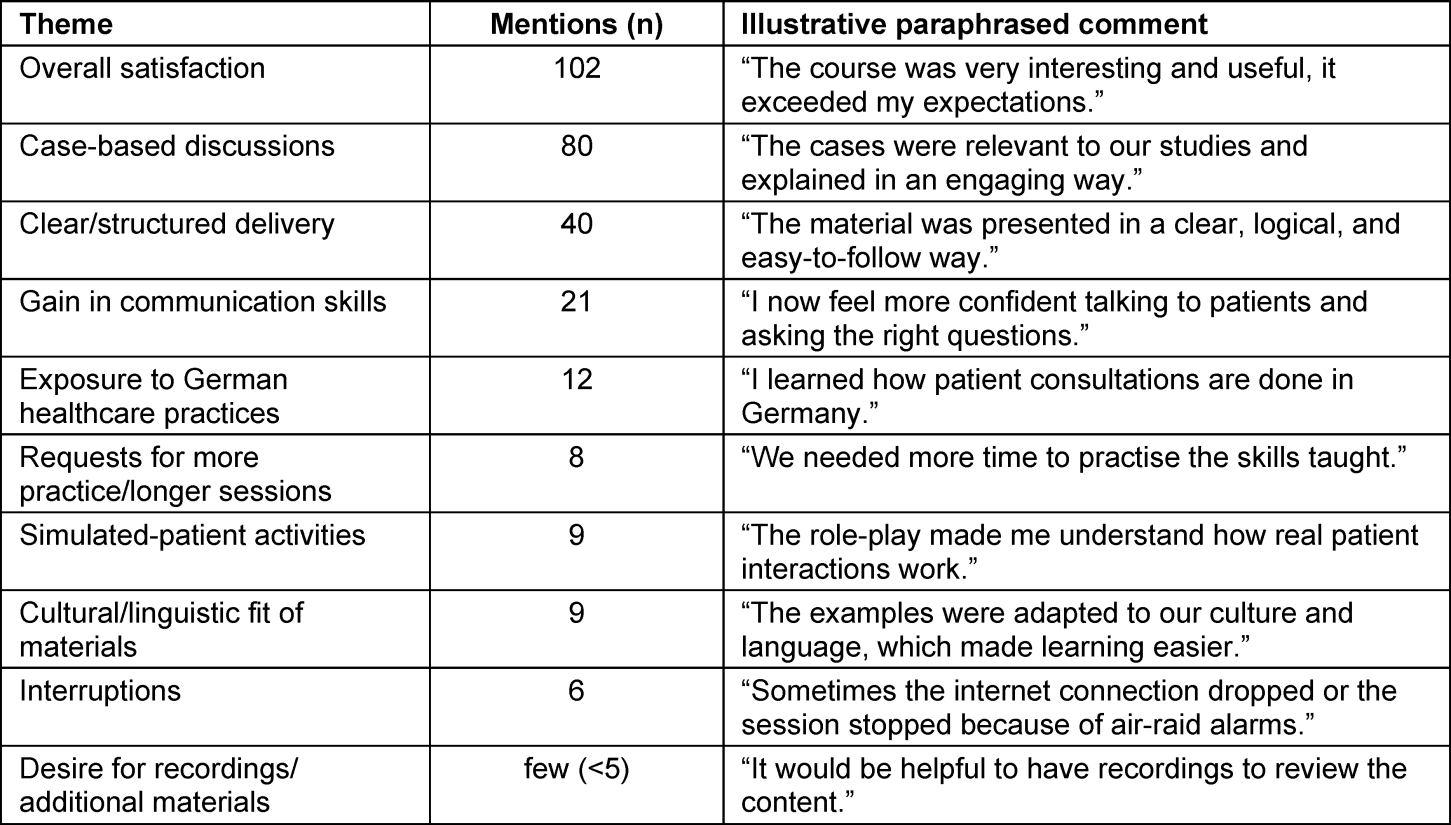
Overview of the most frequently mentioned themes and example statements
